# Supplementary material for: Beyond Motor Deficits: Environmental Enrichment Mitigates Huntington’s Disease Effects in YAC128 Mice
Source: Int J Mol Sci. 2023 Aug 9;24(16):12607. doi: 10.3390/ijms241612607 (PMC10454852; doi:10.3390/ijms241612607)
Supplement: Supplementary file 1 [file ijms-24-12607-s001.zip › ijms-2510581-supplementary.pdf]

Supplementary Files

Table S1. Behavioral results for YAC128 and WT mice at 2- and 4 months of age.

Test/ Age	Evaluation	Two-way ANOVA effects on		
		Genotype	Environment	Genotype x environment interaction
**Pole Test** 2 m	Time to descend	F(1,33)=0.08; p=0.77	F(1,33)=0.06; p=0.81	F(1,33)=2.33; p=0.14
**Pole Test** 4 m	Time to descend	F(1,48)=1.94; p=0.17	F(1,48)=1.71; p=0.20	F(1,48)=0.91; p=0.34
**Open Field** 2 m	Distance traveled	F(1,33)=0.08; p=0.77	F(1,33)=1.71; p=0.20	F(1,33)=0.62; p=0.44
	Center time	F(1,33)=0.02; p=0.87	F(1,33)=0.26; p=0.61	F(1,33)=0.66; p=0.42
**Open Field** 4 m	Distance traveled	F(1,32)=0.03; p=0.86	F(1,32)=0.89; p=0.35	F(1,32)=2.80; p=0.10
	Center time	F(1,32)=1.87; p=0.18	F(1,32)=0.78; p=0.38	F(1,32)=1.29; p=0.26
